# Cholangiocarcinoma presenting as hemobilia and recurrent iron-deficiency anemia: a case report

**DOI:** 10.1186/1752-1947-4-133

**Published:** 2010-05-11

**Authors:** Saif S Ahmad, Faisal TM Basheer, Saad F Idris, Radhakrishnan Hariraj, Rajarathnam Mathialagan, Andrew Douds

**Affiliations:** 1 Trent Cardiac Centre, Nottingham University Hospitals NHS Trust, NG5 1PB, UK; 2 Addenbrooke's Hospital, Cambridge University Hospitals NHS Foundation Trust, Hills Road, Cambridge, CB2 0QQ, UK; 3 Queen Elizabeth Hospital, Gayton Road, King's Lynn, PE30 4ET, UK

## Abstract

**Introduction:**

Iron-deficiency anemia is a relatively common presenting feature of several gastrointestinal malignancies. However, cholangiocarcinoma has rarely been reported as an underlying cause. The association of cholangiocarcinoma with the rare clinical finding of hemobilia is also highly unusual. To our knowledge, this is the first case report of cholangiocarcinoma presenting with acute hemobilia and chronic iron-deficiency anemia.

**Case presentation:**

We report the case of a Caucasian, 84-year-old woman presenting with recurrent, severe iron-deficiency anemia who was eventually diagnosed with intra-hepatic cholangiocarcinoma, following an acute episode of hemobilia. A right hepatectomy was subsequently performed with curative intent, and our patient has now fully recovered.

**Conclusion:**

This is a rare example of hemobilia and chronic iron-deficiency anemia in association with cholangiocarcinoma. We suggest that a diagnosis of cholangiocarcinoma should be considered in patients who present with iron-deficiency anemia of unknown cause, particularly in the presence of abnormal liver function.

## Introduction

Iron-deficiency anemia is a relatively common presenting feature of several gastrointestinal malignancies. However, cholangiocarcinoma has rarely been reported as an underlying cause. Hemobilia describes blood loss from the biliary tract. Its association with cholangiocarcinoma is very uncommon. We describe the case of a patient presenting with recurrent, severe iron-deficiency anemia who, after significant delay, was eventually diagnosed with intra-hepatic cholangiocarcinoma, following an acute episode of hemobilia.

To our knowledge, this is the first case report of cholangiocarcinoma presenting with acute hemobilia and chronic iron-deficiency anemia.

## Case presentation

An 84-year-old British, Caucasian woman was referred to the Gastroenterology department at her local hospital with a six week history of episodic epigastric pain and iron-deficiency anemia. There was no obvious source of blood loss on systemic enquiry and she denied any recent weight loss or change in bowel habit. Her drug and family histories were unremarkable.

One year prior to referral she had been admitted with upper abdominal pain and obstructive jaundice. Ultrasound examination revealed gallstones and a dilated common bile duct. She was subsequently treated with endoscopic retrograde cholangiopancreatography (ERCP) and sphincterotomy. A repeat ERCP showed a bile duct free of stones. However, she continued to suffer with occasional epigastric pain and despite being offered a cholecystectomy she favoured conservative management.

On general examination there was pallor but no jaundice. Her abdomen was soft and non-tender and there was no evidence of any abdominal masses, organomegaly or blood *per rectum*. There was no lymphadenopathy.

Routine investigations confirmed a hypochromic microcytic anemia (hemoglobin 7.7 g/dL) consistent with iron deficiency. Notably, her alkaline phosphatase was raised (394 U/L), but this was attributed to cholelithiasis. She was admitted, was transfused with three units of blood and discharged with oral iron supplementation. An outpatient oesophagoduodenogastroscopy with duodenal biopsy and colonoscopy were subsequently reported as normal. In view of these and with normalization of her hemoglobin and symptomatic improvement, her iron replacement was discontinued.

She was readmitted six months later with a hemoglobin level of 5.5 g/dL and was transfused with four units of blood. There was no evidence of bleeding *per rectum*, and a history of melaena was difficult to establish in view of previous oral iron therapy. A ferritin level of 10 μg/L and iron saturation of 4% indicated severe iron-deficiency anemia, with B12 and folate within normal limits. A bone marrow aspirate showed diminished iron stores. All other routine blood tests were normal except for an alkaline phosphatase remaining above 200 U/L and a low albumin (Table 1).

**Table 1:** Summary of blood investigations

Variable	1st admission (with cholelithiasis) Day 0	2nd admission At 12 months	3rd admission At 18 months	4th admission At 19 months
**Hemoglobin**	**11.0**	**7.6**	**5.5**	**4.7**

Mean corpuscular volume	91.2	77.7	91.0	96

Ferritin	-	-	10	-

Albumin	30	33	27	23

Bilirubin	153	18	13	8

**Alkaline phosphatase**	**778**	**394**	**256**	**270**

Alanine aminotransferase	338	24	19	20

				
**Intervention**	- ERCP + sphincterotomy - Repeat ERCP normal	- Red cell transfusion	- Red cell transfusion - Parenteral iron	- OGD: Hemobilia - CT: Cholangiocarcinoma ⇒ Right hemi-hepatectomy

**Figure 1:**
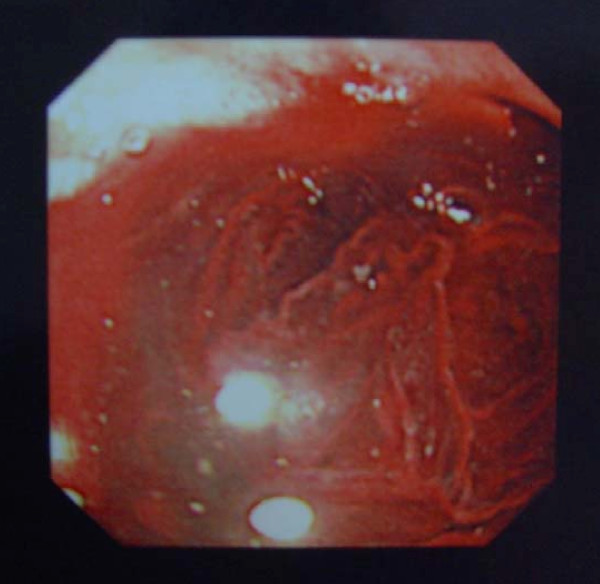
**Oesophagogastroduodenoscopy showing hemobilia**.

Our patient was reluctant to undergo further investigation and her anemia was therefore treated with regular iron infusions. However, she was readmitted for a third time one month later with hemoglobin of 4.7 g/dL, again with no hemodynamic compromise. Three fecal occult blood (FOB) samples were positive. Following further transfusion she underwent a repeat oesophagogastroduodenoscopy, which demonstrated evidence of active bleeding around the ampulla (Figure 1).

**Figure 2:**
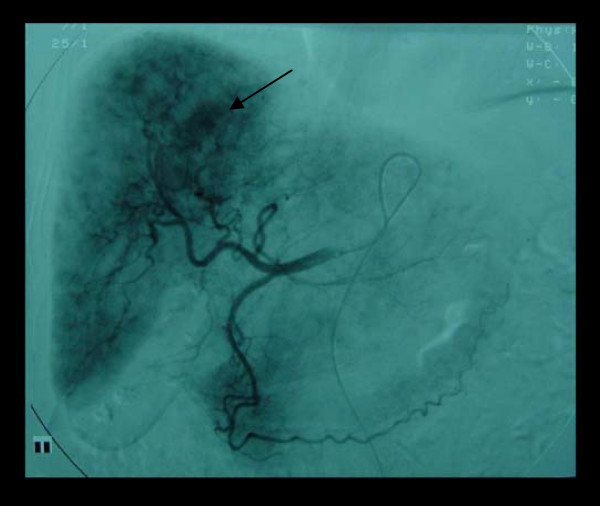
**Superior mesenteric angiogram demonstrating abnormal tumour blush**.

**Figure 3:**
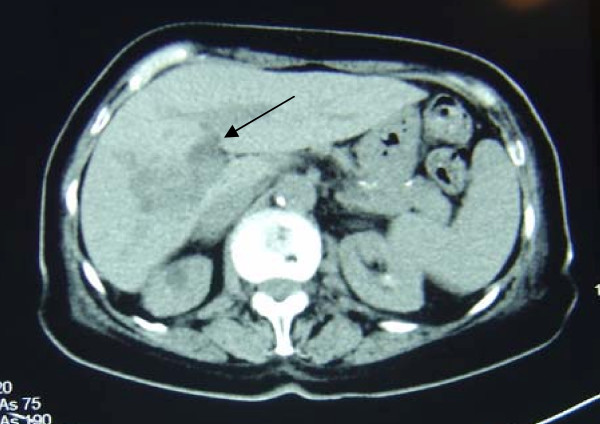
**Abdominal CT scan showing area of low attenuation in right lobe of liver**.

She was then urgently transferred to a tertiary hospital for further management. A superior mesenteric angiogram demonstrated an abnormal tumor circulation in the right lobe of the liver supplied by the right and median hepatic arteries (Figure 2). Subsequent computerized tomography (CT) of the abdomen showed a 2.5 cm lesion in the right lobe of the liver with proximal biliary dilatation: findings consistent with an intrahepatic cholangiocarcinoma (Figure 3).

Suitable treatment options for our patient were then extensively discussed at a multidisciplinary meeting. In view of her age, an angiographic embolization was considered as it would avoid the complications of laparotomy and a major liver resection. However, it was noted that this procedure would be technically difficult and risked embolization of the entire right and median hepatic arteries resulting in occlusion of the cystic artery and subsequent cholecystitis.

Curative surgery was concluded as most appropriate. A right hepatectomy was therefore performed, which included biliary bifurcation with anastomosis of the Roux loop to the remaining left sided ducts. Histology showed appearances of a papillary adenocarcinoma with erosion of surrounding vessels, consistent with the earlier finding of hemobilia. The operation was successful and the patient fully recovered. She remains well and her latest full blood count and alkaline phosphatase (134 U/L) are reassuringly normal.

## Discussion

The extent to which iron-deficiency anemia should be investigated remains a contentious issue [1]. With a UK prevalence of 2% to 5% among adult men [2] and post-menopausal women, it accounts for approximately 100,000 referrals to gastroenterologists per year (4% to 13% of referrals) [3]. Around 10% of these referrals are diagnosed as having primary gastrointestinal tract malignancy. However, almost twice this number remain without a clear diagnosis, and a significant proportion of these patients [4] continue to suffer with recurrent or persistent anemia.

The British Society of Gastroenterology has established guidelines [5] outlining the management of iron-deficiency anemia and highlight the fact that colonic and gastric carcinomata are by far the most common malignant causes [6]. However, in view of the fact that 20% of referrals remain undiagnosed, rarer disorders must be considered in those patients who present with recurrent or persistent anemia with no obvious cause found on routine work up. Ampullary carcinoma is a known, rare cause of iron-deficiency anemia, which given its proximity to the gastrointestinal tract seems entirely logical. However, tumor growth higher in the biliary tree is rarely considered.

Cholangiocarcinoma accounts for 3% of all gastrointestinal cancers and is the second most common primary malignancy of the liver worldwide [7]. Its incidence is increasing and in the UK it has recently overtaken hepatocellular carcinoma as the main cause of death from liver tumours.

To our knowledge, there are no previous case reports of chronic iron-deficiency anemia secondary to cholangiocarcinoma. Acute hemobilia is also a rare presentation of this condition. A literature search revealed only two case reports of cholangiocarcinoma presenting with hemobilia, both based in southeast Asia [8,9], where the disease is more prevalent. This rare clinical finding is most commonly associated with iatrogenic hepatobiliary trauma [10]. The classic triad of hemobilia involves biliary colic, obstructive jaundice and gastrointestinal bleeding, however this occurs in only 30% of cases [11], making its diagnosis difficult to establish.

Other rare causes of hepatopancreatobiliary bleeding have also been reported. Biliary-enteric fistulae should be considered in patients who have had previous surgery. It has also been reported secondary to peptic ulcer disease [12]. Tumors of the pancreas may also present with signs of upper gastrointestinal bleeding [13].

Our patient described above initially presented with obstructive symptoms, and these were ascribed to cholelithiasis, which was appropriately treated by ERCP. However, her subsequent repeated presentations with severe iron-deficiency anemia, persistently elevated alkaline phosphatase and a stone-free bile duct on previous repeat ERCP was inconsistent with this diagnosis. This should have prompted earlier second-line investigations including gamma-glutamyl transferase and FOB. Tumour markers were not checked at any stage. An abdominal CT scan was only performed following an abnormal endoscopy more than six months after representation. Earlier non-invasive imaging may also have been appropriate. This may have avoided repeated hospital admissions and the considerable delay in reaching the diagnosis. The importance of endoscopy in iron-deficiency anemia cannot be overemphasized. A previous normal study may need to be repeated and the ampullary region should also be considered as a source of bleeding.

The mechanism of chronic iron-deficiency anemia in this case may have been secondary to long standing low-grade hemobilia. This is evidenced by the fact that fecal occult blood samples were positive and that there was erosion of surrounding vessels on histology. Anemia of chronic disease may also have been present. Disease progression may have been complicated by acute episodes of blood loss.

It is important to note that the patient was offered surgery despite her age. She has fully recovered from her condition and this illustrates that curative surgery should be considered in all cases and suitable patients should be identified on an individual basis. Newer therapeutic techniques are evolving and notably intraluminal brachytherapy has been used in palliative cases to relieve bleeding and obstruction [14].

## Conclusion

This case illustrates two unusual presentations of intrahepatic cholangiocarcinoma: iron-deficiency anemia and hemobilia. We suggest therefore, that in the presence of recurrent iron-deficiency anemia, non-specific gastrointestinal symptoms and abnormal liver function, a diagnosis of cholangiocarcinoma is considered. Moreover, this case serves to highlight the principle that the persistence of a symptom should be reflected in the persistence with which a clinician aims to establish a clear diagnosis.

## Consent

Written informed consent was obtained from the patient for publication of this case report and any accompanying images. A copy of the written consent is available for review by the Editor-in-Chief of this journal.

## Authors' contributions

SA wrote the initial manuscript, reviewed the patient notes and reviewed recent literature on hemobilia. FB reviewed patient investigations and assisted the literature review. SI reviewed literature on cholangiocarcinoma and assisted writing the manuscript. RH provided expert analysis of the case, RM assisted the literature review and AD fully reviewed the final submission. All authors read and reviewed the final manuscript.

## Competing interests

The authors declare that they have no competing interests.

## References

[B1] Sahay R Scott B Iron deficiency anaemia--how far to investigate? Gut 1993 34 1427 1428 10.1136/gut.34.10.1427 8244114 PMC1374555

[B2] Sayer JM Long RG A perspective on iron deficiency anaemia Gut 1993 34 1297 1299 10.1136/gut.34.10.1297 8244090 PMC1374529

[B3] McIntyre AS Long RG Prospective survey of investigations in outpatients referred with iron deficiency anaemia Gut 1993 34 1102 1107 10.1136/gut.34.8.1102 8174963 PMC1374363

[B4] Yates JM Logan EC Stewart RM Iron deficiency anaemia in general practice: clinical outcomes over three years and factors influencing diagnostic investigations Postgrad Med J 2004 80 405 410 10.1136/pgmj.2003.015677 15254305 PMC1743059

[B5] British Society for Gastroenterology Guidelines for the Management of Iron Deficiency Anaemia 2005

[B6] Rockey DC Cello JP Evaluation of the gastro-intestinal tract in patients with iron-deficiency anemia N Engl J Med 1993 329 1691 1695 10.1056/NEJM199312023292303 8179652

[B7] Khan SA Thomas HC Davidson BR Taylor-Robinson SD Cholangiocarcinoma Lancet 2005 366 9493 1303 1314 10.1016/S0140-6736(05)67530-7 16214602

[B8] Thong-Ngam D Shusang V Wongkusoltham P Brown L Kullavanijaya P Hemobilia: four case reports and review of the literature J Med Assoc Thai 2001 84 3 438 444 11460949

[B9] Bando T Matsuoka J Hashimoto I Oonishi Y Nozawa S Yamagishi F Tsukada K Takahashi H A case of bile duct carcinoma with Haemobilia Japanese J Gastroenterological Surg 2006 39 2 227 231

[B10] Green MHA Duell RM Johnson CD Jamieson NV Haemobilia Br J Surg 2001 88 6 773 786 10.1046/j.1365-2168.2001.01756.x 11412246

[B11] Yoshida J Donahye PE Nyhus LM Haemobilia: review of recent experience with a worldwide problem Am J Gastroenterol 1987 82 448 453 3578223

[B12] La Greca G Grasso E Sofia M Gagliardo S Barbagallo F Complicated duodeno-biliary fistula in bleeding duodenal ulcer: case report an literature review Ann Ital Chir 2008 79 1 57 61 18572741

[B13] Apostolidis S Papavramidis T Zatagias A Michalopoulos A Papadopoulos V Paramythiotis D Harlaftis N Hematemesis, a very rare presentation of solid pseudo-papillary tumors of the pancreas: a case report J Med Case Reports 2008 2 271 10.1186/1752-1947-2-271 18700976 PMC2526092

[B14] Macchia G Costamagna G Morganti AG Mutignani M Giuliante F Clemente G Deodato F Smaniotto D Mattiucci GC Sallustio G Valentini V Nuzzo G Cellini N Intraluminal brachytherapy without stenting in intrahepatic papillary cholangiocarcinoma: a case report Dig Liver Dis 2005 37 8 615 618 10.1016/j.dld.2004.07.022 15890567

